# Supplementary material for: Multigenerational exposure to elevated temperatures leads to a reduction in standard metabolic rate in the wild
Source: Funct Ecol. 2020 Feb 19;34(6):1205–14. doi: 10.1111/1365-2435.13538 (PMC7318562; doi:10.1111/1365-2435.13538)
Supplement: Supplementary file 1 [file FEC-34-1205-s001.pdf]

Functional Ecology

BRITISH ECOLOGICAL SOCIETY

A warmer environment leads to a lower metabolic rate

Natalie Pilakouta, Shaun S. Killen, Bjarni K. Kristjánsson, Skúli Skúlason, Jan Lindström, Neil B. Metcalfe, and Kevin J. Parsons

Climate change is arguably the most significant threat to biodiversity in the 21st century, as rising temperatures are altering environmental conditions and increasing the risk of population extinction. There is thus a pressing need to understand and predict the capacity of populations to adapt to a warming world. Ectothermic animals, such as fishes and reptiles, are particularly vulnerable to rising temperatures, because changes in ambient temperature directly influence their body temperature. A key trait that is likely to determine the capacity of ectotherms to cope with increasing temperatures is metabolic rate.

To understand how metabolic rate may evolve in response to climate change, we took advantage of a powerful 'natural experiment' that provides us with a window into the future. Because of geothermal activity in Iceland, there are many warm lakes in addition to ambient-temperature lakes, which are 5–10°C colder. Our study focused on a small freshwater fish species, the threespine stickleback, which is found in both geothermally warmed lakes (warm habitats) and ambient-temperature lakes (cold habitats). By comparing fish from these warm and cold habitats, we were able to investigate whether being in warm water for many generations leads to a change in metabolic rate.

We found that sticklebacks from warm habitats generally had a lower metabolic rate than those from cold habitats when measured at the same temperature. This suggests that suppressed metabolic rates might

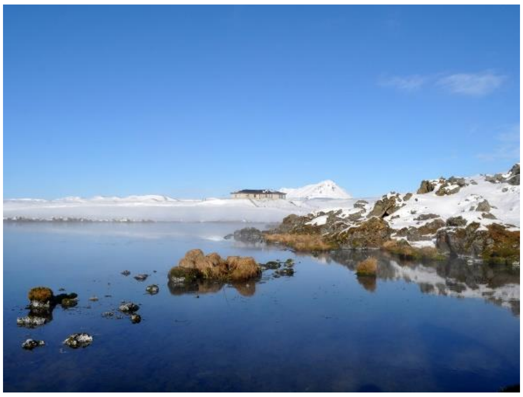

evolve as temperatures rise. Nevertheless, our findings also suggest that gene flow between different thermal habitats might constrain metabolic adaptation.
