# Supplementary material for: Multigenerational exposure to elevated temperatures leads to a reduction in standard metabolic rate in the wild
Source: Funct Ecol. 2020 Feb 19;34(6):1205–14. doi: 10.1111/1365-2435.13538 (PMC7318562; doi:10.1111/1365-2435.13538)
Supplement: Supplementary file 2 [file FEC-34-1205-s002.pdf]

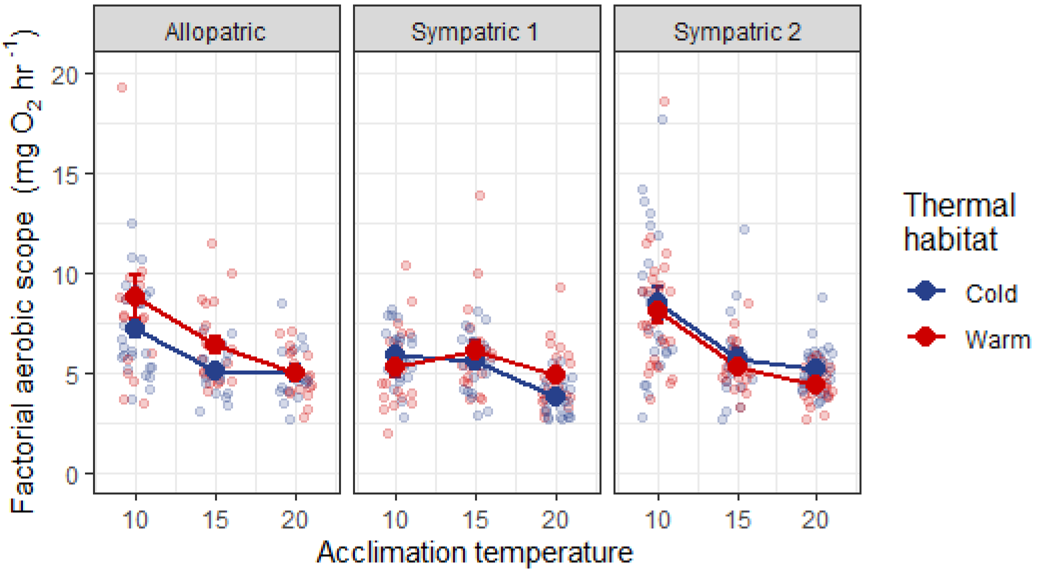

Supplementary Figure 1. Factorial aerobic scope (mg O_2 hr^{-1}) of threespine sticklebacks from cold and warm habitats in Iceland that were acclimated to 10°C, 15°C, or 20°C. Error bars indicate standard errors, and small circles represent individual data points (blue=cold thermal habitat, red=warm thermal habitat). 'Allopatric' refers to Grettislaug and Garðsvatn, 'sympatric 1' refers to Áshildarholtsvatn, and 'sympatric 2' refers to Mývatn.
