# Supplementary material for: Multigenerational exposure to elevated temperatures leads to a reduction in standard metabolic rate in the wild
Source: Funct Ecol. 2020 Feb 19;34(6):1205–14. doi: 10.1111/1365-2435.13538 (PMC7318562; doi:10.1111/1365-2435.13538)
Supplement: Supplementary file 3 [file FEC-34-1205-s003.pdf]

Supplementary Table 1. Samples sizes for standard metabolic rate (SMR), absolute aerobic scope (AAS), and factorial aerobic scope (FAS) measurements at three acclimation temperatures (10 °C, 15 °C, and 20 °C). 'Allopatric' refers to Grettislaug and Garðsvatn, 'sympatric 1' refers to Áshildarholtsvatn, and 'sympatric 2' refers to Mývatn.

Population pair	Thermal habitat	10 °C	15 °C	20 °C
Allopatric population	Warm	21	22	18
	Cold	27	19	15
Sympatric population 1	Warm	20	21	23
	Cold	24	20	26
Sympatric population 2	Warm	30	19	23
	Cold	24	19	31

Supplementary Table 2. Sample sizes for maximum metabolic rate (MMR) measurements at three acclimation temperatures (10 °C, 15 °C, and 20 °C). 'Allopatric' refers to Grettislaug and Garðsvatn, 'sympatric 1' refers to Áshildarholtsvatn, and 'sympatric 2' refers to Mývatn.

Population pair	Thermal habitat	10 °C	15 °C	20 °C
Allopatric population	Warm	24	22	21
	Cold	29	19	15
Sympatric population 1	Warm	22	22	24
	Cold	24	20	29
Sympatric population 2	Warm	35	19	23
	Cold	25	19	33

Supplementary Table 3. Results of general linear model testing the effects of thermal habitat (warm or cold), population pair (allopatric, sympatric 1, or sympatric 2), acclimation temperature (10°C, 15°C, or 20°C), and their interactions on factorial aerobic scope (FAS) in threespine stickleback from six populations in Iceland. Df denotes degrees of freedom. Eta-squared (η^2) represents the percent variance explained by each factor, which was calculated by dividing the sum of squares for each factor by the total sum of squares and multiplying by 100.

	Facultative aerobic scope (FAS)			
	η^2	df	*F*	*P*
Thermal habitat	0.09	1	0.51	0.47
Population pair	3.94	2	11.2	**<0.001**
Acclimation temperature	21.8	1	124	**<0.001**
Mass	1.80	1	10.2	**<0.001**
Thermal habitat × Population pair	0.86	2	2.43	0.089
Thermal habitat × Acclimation temperature	0.09	1	0.54	0.46
Population pair × Acclimation temperature	1.29	2	3.67	**0.026**
Thermal habitat × Population pair × Acclimation temperature	1.64	2	4.66	**0.010**
Error	68.5	385		
